# Where, How, and When: Positioning Posttranslational Modification Within Type 1 Diabetes Pathogenesis

**DOI:** 10.1007/s11892-016-0752-4

**Published:** 2016-05-11

**Authors:** Rene J. McLaughlin, Matthew P. Spindler, Menno van Lummel, Bart O. Roep

**Affiliations:** Department of Immunohematology and Blood Transfusion, Leiden University Medical Center, E3-Q, PO Box 9600, 2300 RC Leiden, The Netherlands; Department of Diabetes Immunology, Diabetes & Metabolism Research Institute, Beckman Research Institute of City of Hope, Duarte, CA 91010 USA; Danish Diabetes Academy, Søndre Blvd. 29, 5000 Odense, Denmark

**Keywords:** Posttranslational modification, Autoimmune disease, Type 1 diabetes, Islet antigens, High-risk HLA

## Abstract

Autoreactive T cells specific for islet autoantigens develop in type 1 diabetes (T1D) by escaping central as well as peripheral tolerance. The current paradigm for development of islet autoimmunity is just beginning to include the contribution of posttranslationally modified (PTM) islet autoantigens, for which the immune system may be ignorant rather than tolerant. As a result, PTM is the latest promising lead in the quest to understand how the break in peripheral tolerance occurs in T1D. However, it is not completely clear how, where, or when these modifications take place. Currently, only a few PTM antigens have been well-thought-out or identified in T1D, and methods for identifying and characterizing new PTM antigens are rapidly improving. This review will address both reported and potential new sources of modified islet autoantigens and discuss how islet neo-autoantigen generation may contribute to the development and progression of T1D.

## Introduction

Type 1 diabetes (T1D) is characterized as an autoimmune disease resulting from the loss of immune tolerance to beta cell autoantigens. However, if posttranslational modification (PTM) of beta cell proteins generates neo-autoantigens that the immune system is ignorant to, as the antigens may not have been present during thymic selection, then T1D could be a result of the immune system responding to essentially “foreign” proteins created by PTM. This may explain some instances of T1D development but probably not all, as a diverse immunological heterogeneity exists between patients in terms of immunogenetic background, islet autoantibodies, and islet autoreactive T cells [1]. This heterogeneity has important implications for guiding therapy, as the degree of cellular islet autoimmunity predicted the clinical outcome of both hematopoietic stem cell therapy and islet transplantation [2–4]. In these studies, the patient’s immune signature correlated with differential responsiveness to immune therapy, underscoring the importance of deciphering disease heterogeneity between patients. Exploring PTM has revealed new immunologically and clinically relevant neo-epitopes as targets for autoreactive T cells, has increased our understanding of disease heterogeneity, and may allow for more personalized therapeutic interventions [5••, 6].

The human proteome plays a role in shaping proper immune discrimination during thymic selection. Whereas the genome comprises 20–25,000 genes [7], the proteome is estimated to encompass over one million proteins [8]. Changes at the mRNA and transcriptional level increase the size of the transcriptome in relation to the genome [9, 10]; following this expansion, the many different PTMs increase the complexity of the human proteome relative to the genome and the transcriptome. PTM is a broad term encompassing many natural posttranslational processes within cells and tissues. These include covalent attachment of small functional molecules (e.g., ubiquitination and phosphorylation), or chemical modifications of amino acids within proteins. Nature supplies 20 amino acids as the building blocks of human proteins, and this number increases to more than 140 building blocks after accounting for PTM through enzymatic modifications (e.g., deamidation, citrullination, glycosylation) and nonenzymatic (spontaneous) modifications (e.g., methylation, carbamylation, oxidation, nitration). In T1D, both citrullinated and deamidated autoantigens have been identified (Table 1), indicating a role for peptidylarginine deiminases (PADs) and tissue transglutaminase (tTG), respectively, in the generation of islet neo-autoantigens.

**Table 1:** Posttranslational modifications in human autoimmune diseases

Type of modification	Antigen(s)	Relevance in autoimmune disease	References
Citrullination Methylation Palmitoylation Acetylation Sulfation	Myelin basis protein (MBP), myelin proteolipid protein (PLP), P-selectin glycoprotein ligand 1 (PSGL-1)	Multiple sclerosis	[62, 63] [64, 65] [66, 67] [68, 69] [70]
Deamidation	Gluten (gliadin)	Celiac disease	[71, 72]
Citrullination Carbamylation Glycosylation	Vimentin, vinculin, histones, apolipoproteins, fibrinogen, Ig	Rheumatoid arthritis	[73, 74] [75, 76] [75]
Deamidation Disulfide bridges Citrullination Oxidative modification	Preproinsulin, GAD65, collagen type II	Type 1 diabetes	[5••] [6] [29] [46, 47, 48•, 77]
Oxidative modification Sialylation	Beta2-glycoprotein 1	Antiphospholipid syndrome	[78, 79] [80]
Phosphorylation Deimination	Cytoplasmic proteins (e.g. Ro/SSA, La/SSB, nucleosomal DNA, histones)	Systemic Lupus Erythematosus	[81] [82] [83]

The involvement of PTM in the pathogenesis of T1D will be discussed by looking at where the cells and tissues regulate protein modification, how the modifications occur, and when during the disease process the modifications are important. This will identify avenues that the T1D community can follow in order to better understand PTM and start developing approaches for disease monitoring and therapeutic intervention.

## Where: Dialogue Between Beta Cells and the Immune System

A direct association between beta cell destruction was established with the discovery of islet autoreactive CD8 T cells in insulitic lesions from patients with T1D [11]. Islets from patients with T1D also had hyper-expression of HLA class I molecules, indicating that during insulitis, beta cells could be active in their own demise by becoming easier targets for pathogenic CD8 T cells [11]. Beta cells could be passive victims to autoimmune destruction (“Homicide” model) and/or actively contribute to their own demise (“Suicide” model), as was first conceived by Bottazzo [12]. Supporting the “beta cell suicide” model is data showing production of the chemokine CXCL10 within distressed islets and expression of the cognate receptor CXCR3 on CD8 T cells in insulitic lesions in patients with T1D, indicating that the pancreas can actively recruit pathogenic CD8 T cells and communicate with the immune system [13].

When considering the impact of PTM on the development and progression of T1D, it is important to consider the location and local activity of the modifying enzymes involved. PTM in celiac disease (CD) occurs in the intestinal lumen at the site where key substrates, such as gliadin peptides, are concentrated. Tissue transglutaminase (tTG) activity is increased in the mucosal epithelium of patients with CD [14], and the ensuing cross talk between immune cells in the lamina propria during CD has been well studied [15]. In human islets, tTG is active during insulin secretion, acting on cytosolic, mitochondrial, and nuclear substrates [16]. Therefore, islets have the potential to generate neo-autoantigens through tTG-mediated deamidation. We recently confirmed that human islets generate neo-autoantigens; an inflammatory stimulus resulted in deamidation of the proinsulin C-peptide [17•]. T cells reactive to this deamidated C-peptide were found in patients with T1D [5••], linking neo-antigen generation in human islets with the induction of autoreactive T cells. tTG is also present in cells of the myeloid lineage [18]. Direct vesicular transfer of islet material to resident APC has been demonstrated in both mice and humans, suggesting that islet proteins can be deamidated by tTG during antigen processing within the APC [19]. Recent results extend these findings to T1D, as dendritic cells (DC) pulsed with native islet antigen 2 (IA-2) naturally processed and presented deamidated peptides from IA-2 [17•]. Thus, it appears that tTG activity in both islets and DC contributes to islet neo-epitope formation. Besides the diverse subcellular localization of tTG, it is also a secreted, extracellular protein important for cell-extracellular matrix interactions [20, 21]. However, the contribution of extracellular tTG to the deamidation of islet proteins within the pancreas has not yet been investigated. PAD expression and activity have not been investigated in human islets, but antigen presenting cells (APC), such as human monocytes and macrophages, contain both PAD2 and PAD4 transcripts and active PAD2 and PAD4, indicating that cells of the monocytic lineage have the potential to citrullinate self-proteins [22]. Mouse APC from both splenic and thymic tissue can citrullinate and present citrullinated peptides to activate epitope-specific CD4 T cells, a process requiring autophagy [23]. While not conclusive, these studies indicate that islet protein citrullination may occur in APC within human islets or in the draining lymph nodes.

## How: Mechanisms of Posttranslational Modification in Type 1 Diabetes

Data on PTM of islet autoantigens is emerging (Table 1), and the crossover with other autoimmune diseases indicates that PTM may have clinical relevance for T1D. HLA binding is a key factor in the selection of processed (neo)epitopes presented by APC and beta cells. To bind HLA molecules, processed peptides must conform to specific HLA binding motifs. In T1D, different HLA molecules associated with disease have distinct peptide-binding preferences [24, 25]. Differences in HLA peptide-binding motifs and the ability to generate stable peptide-HLA complexes likely explain the striking differences in the genetic risk associated with different HLA genotypes. For example, the peptide binding repertoires of the molecular variants of the high-risk HLA-DQ2, DQ8, and DQ2/8 genotypes showed strong preferences for binding peptides with negatively charged residues [26]. As such, the genetic association of T1D with HLA-DQ can potentially be explained by the deamidating function of tTG, which creates more negatively charged peptides that can be exceptionally potent HLA-DQ binders. Indeed, deamidation of a naturally processed and presented PPI peptide enhanced binding to HLA-DQ2/8 molecules and specifically to DQ8*trans*, a heterodimeric HLA molecule composed of the DQ2 alpha and DQ8 beta chains, and patient T cell responses (interferon gamma) were increased after stimulation with the deamidated PPI peptide [5••]. Deamidation of chromogranin A, a recently discovered antigen in T1D, also increased T cell interferon gamma in patients with T1D [27]. Interestingly, the HLA-DQ8*trans* molecule is also implicated in CD pathogenesis, as demonstrated by T cell cross-reactivity between HLA-DQ8 and DQ8*trans* [28]. Citrullinated GAD65 peptides displayed enhanced binding to HLA-DR4 and were recognized by autoreactive CD4 T cells isolated from patients with T1D [29]. Citrullinated glucose-regulated protein 78 was identified as a modified autoantigen in murine beta cells and was a target for autoreactive T cells in mice [30]. Citrullination could be increasing the binding affinity to predisposing HLA molecules by making the peptide more acidic, resulting in increased immunogenicity.

The common factor linking these modifications is the increase in HLA binding affinity that results from PTM. As such, stringent selection of a high-affinity TCR repertoire against modified islet proteins is likely to play a role in T1D pathogenesis. This has been shown in CD, where autoreactive CD4 T cells isolated from patients with CD expressed an HLA-DQ2 or HLA-DQ8-restricted, gluten peptide-specific immunodominant TCR, with high avidity for HLA-DQ-gluten peptide complexes. This concept might also bear relevance in RA pathogenesis [31–33].

## When in the Disease Process Are PTM Important?

**Fig. 1:**
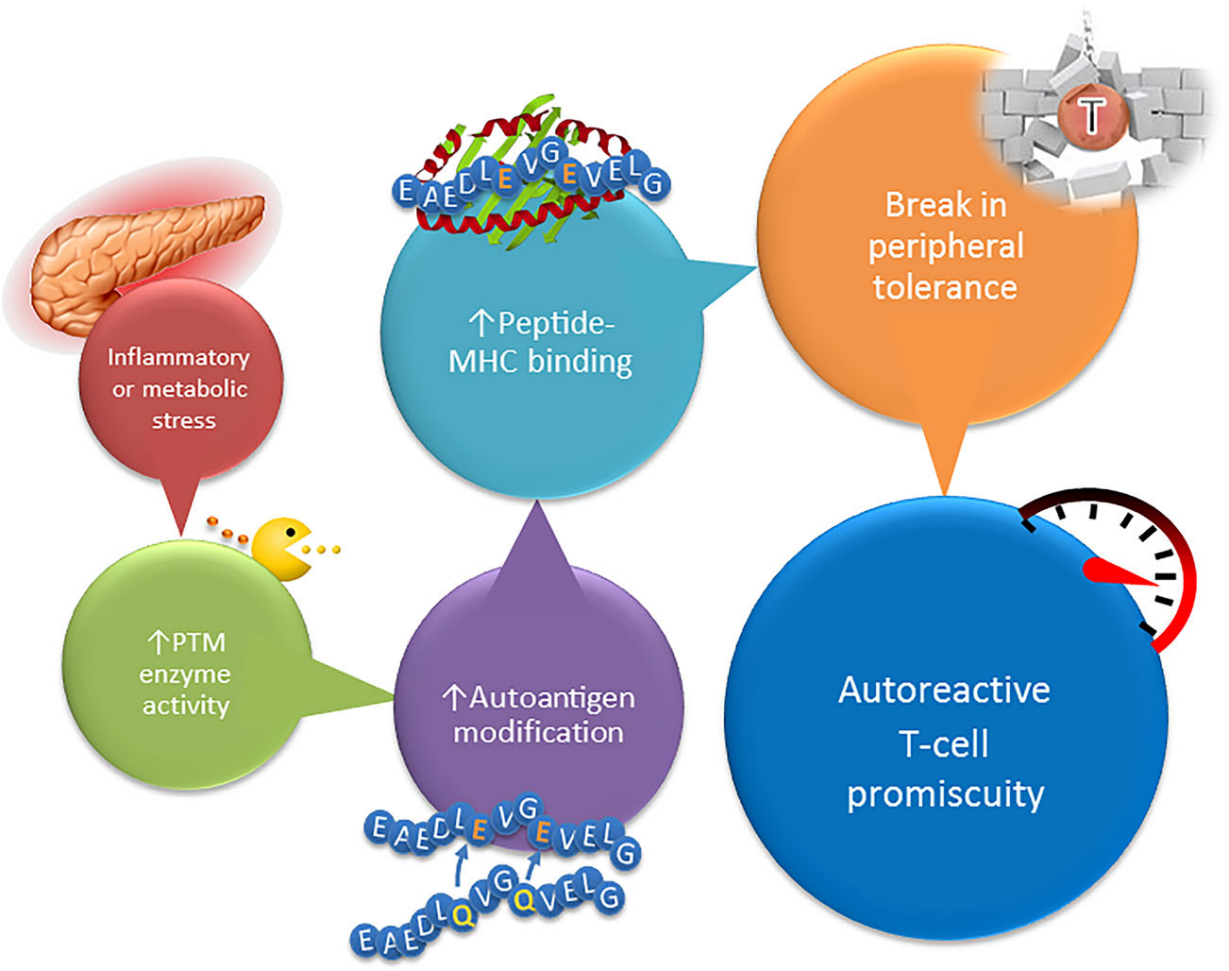
Connecting posttranslational modification of islet proteins with the development of type 1 diabetes. This model begins with an as yet undefined environmental stressor. Lead candidates include metabolic stress, via changes in blood glucose concentration, and inflammatory stress, perhaps through viral infection or the close links between the pancreas and the gut. Stress results in a surge in PTM enzyme activity in islets and APC. This surge creates a larger pool of modified proteins, increasing the opportunity for modified peptides to be presented within the islet immune compartment. The genetic predisposition becomes important, as the HLA molecules in question (DQ2 and DQ8) are exceptionally adept at binding deamidated epitopes. If the modified epitopes are treated as “foreign” by the immune system, then an immune response will ensue. This leads to a break in peripheral tolerance to unmodified islet antigens via promiscuous T cells that are able to recognize both their cognate deamidated epitope and the native sequence. Beta cells readily present unmodified antigens, so the autoreactive response quickly becomes destructive, resulting in overt diabetes

Given the overlapping HLA class II susceptibility haplotypes in CD and T1D, and the growing evidence that tTG can modify beta cell antigens, it is reasonable to frame our discussion of when PTM occurs in T1D after our understanding of CD, where deamidation of gluten peptides by tTG results in preferential loading onto predisposing HLA-DQ molecules and the activation of pathologic CD4 T cells [34]. In this model, deamidation and neo-epitope formation are required for disease and precede autoimmunity. In the case of T1D, it is conceivable that a precipitating event such as nonspecific inflammation, metabolic stress, smaller pancreas size [35], or a viral infection [36] activates modifying enzymes such as tTG in beta cells and within tissue resident APC, thereby generating modified beta cell proteins that are recognized by the immune system (Fig. 1). The link between CD and T1D goes beyond HLA-DQ susceptibility as a gluten-free diet was shown to prevent diabetes in the NOD mouse model and even resulted in remission in a 6-year-old child with T1D [37–40]. These positive effects, resulting from removing gluten from the diet, may relate to the impact of gluten on the composition of the intestinal microbiota, leading to increased gut permeability and subsequent activation of innate immunity and autoimmunity in the gut [37, 41, 42]. A relation between the intestinal microbiome and PTM of islet autoantigens, in particular through activation of tTG, remains to be established. Emerging reports in human T1D indicate that modified antigens are preferentially recognized over their native counterparts. T cells derived from a T1D subject expanded in response to a modified insulin peptide containing a disulfide bond but did not elicit a response in healthy subjects [6]. CD4 T cells specific for deamidated and citrullinated GAD65 peptides were found at higher frequencies in patients compared to HLA-matched controls [29]. Our own observations demonstrated that T cell responsiveness to the deamidated versus the native form of a proinsulin peptide was greatly increased in recent onset patients [5••]. Given the low probability for thymic deletion of PTM protein-reactive T cells and the higher binding affinity of deamidated autoantigens for the predisposing HLA-DQ molecules, it is tempting to propose that modified beta cell antigens are being regarded as foreign by the immune system.

Intriguingly, attempts to generate T cells lines to native proinsulin failed, whereas T cell lines against deamidated proinsulin peptides expanded rapidly and, once generated, cross-reacted with native proinsulin, suggesting that priming may occur against modified self-protein that spreads to the native proteome [5••]. This evidence suggests that immune recognition of native autoantigens is preceded by recognition of modified autoantigens and requires a degree of T cell promiscuity. Exactly when the onset and timeline of diabetes development is influenced by PTM is still unclear, but it is interesting to speculate that the degree to which an individual responds to modified antigens may correlate with an accelerated clinical manifestation of diabetes.

Which modified antigens are most immunogenic and when they become important for development of autoimmunity is also unclear. Insulin and GAD65 antibodies are usually the first antibodies detected in patient serum, and modifications of these antigens may play a particularly important role in disease initiation. Indeed, insulin and GAD65 exist in modified forms that elicit stronger immune responses in patients with T1D than the native epitopes [5••, 6, 29]. Individuals who are positive for multiple autoantibodies have an increased risk of developing diabetes and generally are diagnosed at an earlier age and require more aggressive insulin regimens [43]. Measuring autoantibodies specific for modified proteins is already used in RA as a highly sensitive diagnostic tool even prior to RA onset [44, 45]. Intriguingly, antibodies have been detected in serum of patients with T1D recognizing GAD65 modified by reactive oxygen species [46] and antibodies recognizing oxidative-modified collagen type II [47]. More recently, antibodies to oxidative modified insulin were detected in patients with T1D that were negative for the presence of insulin autoantibodies [48•]. If PTM of islet proteins preludes T1D development, the presence of autoantibodies directed against neo-antigens in patients with T1D may provide an earlier and more robust biomarker for T1D development.

## Clinical and Therapeutic Relevance of PTM

Current methods for predicting T1D during the preclinical phase rely on serum autoantibody levels. These serological markers have proved successful at identifying individuals most at risk, but only a subset of islet autoantibody-positive individuals progress to clinical diabetes [49]. Currently, there are no biomarkers to distinguish those individuals that develop disease versus those that remain asymptomatic. It is also becoming increasingly clear that T1D is a heterogeneous disease, and classical serological markers have not proven useful in stratifying the high-risk population [50, 51]. Furthermore, the relatively late appearance of classical autoantibodies in diabetes progression precludes any information on the timing of disease initiation [52].

Autoantibodies specific for modified islet proteins may address these limitations and be a more relevant clinical tool. If autoantibodies against neo-autoantigens mark events closer to the initiation of islet inflammation than classical autoantibodies, there could be an opportunity to intervene prophylactically rather than therapeutically to prevent the break in immune tolerance. The specificity for a particular neo-autoantigen could also help to characterize various forms of islet autoimmunity and help inform decisions on which therapeutic strategies would prove most promising for specific individuals. If clinical symptoms correlate with both the immunogenicity of a particular modification and the degree to which this modification occurs, measuring PTM could be used to predict those high-risk individuals most likely to progress to clinical diabetes.

Evidence of autoantibodies to neo-autoantigens is increasing; however, advances in mass spectrometry open the possibility of detecting modified molecules directly in patient serum. Analysis of the serum proteome for biomarker development has been limited by the extremely low abundance of relevant circulating proteins [53]. Advances in sample preprocessing have improved the detection limit, however, making it feasible to quantify changes in the serum proteome. This approach was used to identify increases in the levels of serum amyloid protein A (SAA), C-reactive protein (CRP), adiponectin, and insulin-like growth factor binding protein 2 in patients with T1D [53]. These innovations in proteomics and the recent advances in our understanding of PTM relevant to T1D warrant using the serum proteome to track molecular changes of islet peptides throughout the progression of T1D. Considering PTM of islet proteins alongside markers of beta cell stress and/or destruction will vastly improve the resolution of preclinical disease progression, giving us the means to link autoimmune events with changes in islet pathology in patients.

PTM also hold promise for efforts aimed at (re)establishing peripheral tolerance to islet autoantigens. Peptide immunotherapy is currently progressing from preclinical models into phase I trials, based on observations that tolerogenic DC (tolDC) pulsed with islet peptide induce antigen-specific immune tolerance [54–56]. These therapeutic strategies could easily extend to include PTM islet peptides. Interestingly, how the peptide was delivered produced a differential regulatory response, as a peptide vaccine approach induced regulatory T cells, while peptide presented by to lDC resulted in IL-10-mediated regulation [54]. Recent results indicate that HLA-DQ2/8 restricted DC process and present both native and deamidated peptides derived from native islet autoantigens [17•]. This suggests that including PTM in a vaccine approach might be redundant, since introducing a native peptide may be sufficient to target T cells specific for both the native and deamidated forms. The fact that DC presented deamidated epitopes suggests this might be an additional regulatory mechanism for promoting peripheral tolerance and opens up the possibility that this process is defective in some patients [57].

There are still many questions on how best to exploit our emerging knowledge of PTM in T1D. For instance, which peptides should be considered for immunotherapy? Which conditions activate modifying enzymes that contribute to PTM? The infamous chicken versus the egg issue must also be considered. Do metabolic stress [58], reduced pancreas size [35], viral infection of beta cells [36], or perhaps even insulitis [11] contribute to PTM? In these cases, PTM may be a consequence, rather than a cause of islet autoimmunity. As our understanding of the peptide-MHC-TCR interaction grows, we could envision a targeted vaccine strategy that is based on an individual’s HLA genotype. Of course, it is also possible that different PTMs trigger autoimmunity in different individuals, even if they are HLA matched. Additional factors such as age, sex, and environmental contact could all influence the effectiveness of a vaccination regimen. The length of the peptide used for inducing tolerance should also be scrutinized carefully. If a peptide sequence is too short, it may directly associate with MHC molecules and evade internalization. This may be important considering our observation that DC can modify internalized peptide. Thus, the use of synthetic long peptides, such as those used for cancer therapy, may be a likely starting point [59].

It is also unclear whether different PTMs are important at different stages of disease or if PTMs collectively are most important at disease initiation. Despite these uncertainties, further investigation of PTM in the context of T1D will increase our understanding of disease pathogenesis, which will undoubtedly stimulate new diagnostic and therapeutic concepts for intervention and prevention.

## Challenges and Conclusions

To understand the pathogenesis of T1D, the postulate of immunological “ignorance” against neo-antigens as an underlying mechanism should be further investigated. It is essential to think “out of the box,” and several issues should be considered when investigating PTM of islet proteins and their role in immune activation in T1D.

Proteomic studies on pancreatic islets are of indispensable value to decipher PTM in the search toward therapeutic targets in T1D. Currently, mass spectrometry is the state-of-the-art technique to profile proteins in biological samples, and in the last decade, mass spectrometers have been improved in terms of sensitivity, resolution, and measurement speed. Identification of proteins in biological samples involves screening the obtained peptide spectra with peptide sequence databases (e.g., Mascot). We recommend adjusting the rules of engagement regarding proteome analysis for confident identification of PTM peptides. It is important to be preemptive in terms of which PTM to look for, as conventional peptide sequence screening does not include variable PTM, such as deamidation and citrullination. In this context, the Mascot score may have less importance, as PTM peptides might not be as abundant, meaning that identified PTM peptides with a low Mascot score should not be discounted too quickly. It may also be necessary to include individual disease-specific sequences, such as new splice variants and cross-linked products from endopeptides, if they are not yet included in databases such as Mascot.

Unraveling patient heterogeneity and creating a T1D “immunological barcode” would improve the efficacy of single and combination (synergistic) therapies for specific groups of patients (personalized medicine). Appreciation of disease heterogeneity has been underscored by finding that insulitic lesions had varying degrees of islet infiltration and beta cell loss across affected organs [11]. Thus, considering the immunological heterogeneity and polygenetic nature of T1D, it is plausible that generation of neo-antigens does not take place uniformly, either within the pancreatic islets of one individual or amongst different individuals. Similarly, the same neo-antigens may not be equally important in initiating or exacerbating disease among different genetically predisposed individuals.

Biomarkers worthy of investigation include autoantibodies directed against neo-antigens. Autoantibodies have already proven to be useful in T1D research, autoantibodies against native insulin were associated with differential outcome of immunotherapy [60], and pancreas transplant recipients with autoantibodies to native GAD65 were better served with thymoglobulin induction therapy than daclizumab to reduce their risk of rejection episodes [61]. Autoantibodies to posttranslationally modified insulin have been detected in patients with T1D, where autoreactivity to oxidized insulin in patients with T1D was more prevalent than native insulin autoantibodies [48•]. However, additional studies are required to further elucidate the role of autoantibodies against PTM antigens in T1D pathogenesis and their possible role as early biomarkers for disease onset. It would be of interest to evaluate whether autoantibodies against neo-antigens are present in large cohorts of patients with T1D (recent-onset, longstanding, longitudinal) and in patients who are classified as autoantibody negative to the existing biomarkers (insulin, GAD65, Znt-8).

Ultimately, the real challenge is for researchers to constantly rethink the current dogma around T1D pathogenesis. The inclusion of PTM and neo-antigens will hopefully lead to a better understanding of autoimmune activation, disease progression, and regulation, validation of novel biomarkers, and development of successful immune therapies.
